# Booster vaccination with Ad26.COV2.S or an Omicron-adapted vaccine in pre-immune hamsters protects against Omicron BA.2

**DOI:** 10.1038/s41541-023-00633-x

**Published:** 2023-03-16

**Authors:** Maarten Swart, Joan van der Lubbe, Sonja Schmit-Tillemans, Ella van Huizen, Johan Verspuij, Ana Izquierdo Gil, Ying Choi, Chenandly Daal, Aditya Perkasa, Adriaan de Wilde, Erwin Claassen, Rineke de Jong, Katrin E. Wiese, Lisette Cornelissen, Marieke van Es, Marjolein van Heerden, Eleni Kourkouta, Issam Tahiri, Michel Mulders, Jessica Vreugdenhil, Karin Feddes - de Boer, Leacky Muchene, Jeroen Tolboom, Liesbeth Dekking, Jarek Juraszek, Jort Vellinga, Jerome Custers, Rinke Bos, Hanneke Schuitemaker, Frank Wegmann, Ramon Roozendaal, Harmjan Kuipers, Roland Zahn

**Affiliations:** 1 Janssen Vaccines & Prevention, Leiden, The Netherlands; 2 Wageningen Bioveterinary Research, Wageningen University & Research, Lelystad, The Netherlands; 3 Janssen Research and Development, Preclinical Sciences and Translational Safety, Beerse, Belgium; 4 Dekking Consultancy, Leiden, The Netherlands

**Keywords:** Vaccines, Infectious diseases

## Abstract

Since the original outbreak of the SARS-CoV-2 virus, several rapidly spreading SARS-CoV-2 variants of concern (VOC) have emerged. Here, we show that a single dose of Ad26.COV2.S (based on the Wuhan-Hu-1 spike variant) protects against the Gamma and Delta variants in naive hamsters, supporting the observed maintained vaccine efficacy in humans against these VOC. Adapted spike-based booster vaccines targeting Omicron variants have now been authorized in the absence of human efficacy data. We evaluated the immunogenicity and efficacy of Ad26.COV2.S.529 (encoding a stabilized Omicron BA.1 spike) in naive mice and in hamsters with pre-existing immunity to the Wuhan-Hu-1 spike. In naive mice, Ad26.COV2.S.529 elicited higher neutralizing antibody titers against SARS-CoV-2 Omicron BA.1 and BA.2, compared with Ad26.COV2.S. However, neutralizing titers against the SARS-CoV-2 B.1 (D614G) and Delta variants were lower after primary vaccination with Ad26.COV2.S.529 compared with Ad26.COV2.S. In contrast, we found comparable Omicron BA.1 and BA.2 neutralizing titers in hamsters with pre-existing Wuhan-Hu-1 spike immunity after vaccination with Ad26.COV2.S, Ad26.COV2.S.529 or a combination of the two vaccines. Moreover, all three vaccine modalities induced equivalent protection against Omicron BA.2 challenge in these animals. Overall, our data suggest that an Omicron BA.1-based booster in rodents does not improve immunogenicity and efficacy against Omicron BA.2 over an Ad26.COV2.S booster in a setting of pre-existing immunity to SARS-CoV-2.

## Introduction

In response to the SARS-CoV-2 (COVID-19) pandemic, multiple spike-based vaccines were rapidly and successfully developed. These vaccines showed high efficacy against COVID-19 and have been deployed worldwide. Janssen developed the Ad26.COV2.S (Jcovden^TM^) vaccine, which is a replication-incompetent human adenovirus type 26 (Ad26) vector^1^ encoding a stabilized pre-fusion SARS-CoV-2 spike protein based on the Wuhan-Hu-1 isolate^2^. A phase 3 clinical trial demonstrated that Ad26.COV2.S was 74.6% efficacious against severe-critical COVID-19^3^. The Ad26.COV2.S COVID‐19 vaccine was granted emergency use authorization in the US and marketing authorization in the European Union as well as in more than 50 other countries.

Several rapidly spreading SARS-CoV-2 variants of concern (VOC) have evolved since the initial introduction of the virus into humans, such as the Beta, Gamma, and Delta variants. The emergence of the Omicron BA.1 variant (initially named B.1.1.529) and Omicron subvariants such as BA.2, BA.4, BA.5, and BQ.1, sparked concerns about vaccine efficacy as it carries an unparalleled number of mutations in the spike protein. BA.1 carries 15 mutations compared with Wuhan-Hu-1 in its receptor binding domain (RBD), which is an immunodominant target for neutralizing antibodies^4^ that are important for the protection against SARS-CoV-2 infection^5^. These mutations enable Omicron BA.1 to extensively evade neutralizing antibodies induced by previous infections and vaccination^6,7^. Nevertheless, a real-world evidence study showed a homologous boost with Ad26.COV2.S administered 6–9 months after primary vaccination provided more than 80% protection against hospitalization during the Omicron BA.1 wave in South Africa^8^. Vaccine protection against severe disease despite low or undetectable Omicron neutralizing antibody titers may be maintained by conserved cellular immunity^9–11^ and non-neutralizing antibodies with Fc-effector functions^12^ across SARS-CoV-2 variants. However, it cannot be excluded that next to an antibody increase due to anamnestic responses after infection, low or undetectable neutralizing antibody titers in vitro neutralization assays contribute to protection in vivo^5,13^.

Here, we show that an Omicron BA.1-based vaccine candidate (Ad26.COV2.S.529) induces higher Omicron BA.1 and BA.2-neutralizing antibody titers than Ad26.COV2.S in naive mice. However, a booster with Ad26.COV2.S, Ad26.COV2.S.529 or a combination of the two vaccines provided comparable protection against Omicron BA.2 in hamsters with pre-existing immunity to Wuhan-Hu-1 spike.

## Results

### Ad26.COV2.S protects against the SARS-CoV-2 B.1.22, Gamma and Delta variants

**Fig. 1:**
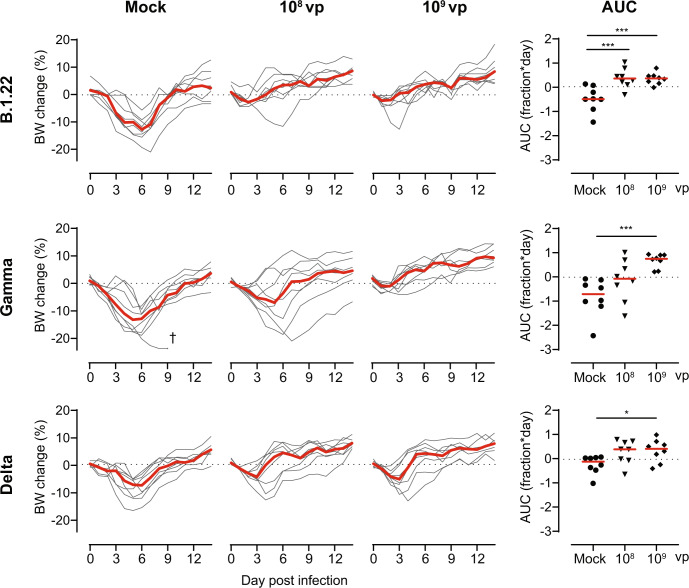
Ad26.COV2.S protects against bodyweight loss upon SARS-CoV-2 B.1.22, Gamma and Delta challenge. Hamsters were vaccinated with formulation buffer (mock), 10^8^ or 10^9^ vp Ad26.COV2.S at day −28 (*n* = 8 per group). The animals were intranasally challenged with 10^3^ TCID_50_ SARS-CoV-2 B.1.22, 10^4^ TCID_50_ SARS-CoV-2 Gamma, or 10^4^ TCID_50_ SARS-CoV-2 Delta on day 0 and followed up till day 14. Individual bodyweight (BW) traces are shown as a percentage compared with the pre-challenge weight. The area under the curve (AUC) is expressed as fraction*day compared with the pre-challenge weight. Red lines indicate group medians. † indicates a humane endpoint was reached. Comparisons to the mock group were performed by ANOVA with a post hoc *t* test with a twofold Bonferroni correction. Statistical differences are indicated by asterisks: *P < 0.05, ***P < 0.001.

It has previously been shown that a vaccine-dose of 10^9^ and/or 10^10^ viral particles (vp) Ad26.COV2.S protects hamsters against challenges with the ancestral A (USA-WA1/2020), B.1 (D614G), and Beta variants^14–16^. Here, we further characterized the protection against the SARS-Cov-2 variants B.1.22 (Supplemental Table 1), Gamma, or Delta in hamsters 4 weeks after vaccination with 10^8^ or 10^9^ vp of Ad26.COV2.S or mock-control. Severe weight loss was observed in mock-vaccinated hamsters after challenge with SARS-CoV-2 B.1.22 (median peak relative weight loss 13.1%) and Gamma (median peak relative weight loss 14.0%), while weight loss was slightly less severe after SARS-CoV-2 Delta challenge (median peak weight loss 9.1%; Fig. 1). The area under the curve (AUC) for relative bodyweight loss was calculated over the entire 14-day follow-up after infection to assess the protection against weight loss in vaccinated animals. Compared with mock-vaccinated animals, animals vaccinated with 10^9^ vp Ad26.COV2.S showed almost complete protection against weight loss upon challenge with SARS-CoV-2 B.1.22, Gamma, and Delta. Although 10^8^ vp Ad26.COV2.S also protected against weight loss upon SARS-CoV-2 B.1.22 challenge, protection against SARS-CoV-2 Gamma and Delta did not reach statistical significance.

Moreover, we evaluated activity loss using activity wheels as a surrogate marker of clinical health^17^. The activity was severely diminished from day 2 up to day 7 after the challenge in all mock-vaccinated animals (Supplemental Fig. 1). Animals were vaccinated with 10^9^ vp Ad26.COV2.S regained pre-challenge activity levels faster after infection with either SARS-CoV-2 B.1.22, Gamma, or Delta than mock-vaccinated animals, which was reflected by a higher AUC for activity change over the entire 14-day follow-up after infection with SARS-CoV-2 B.1.22 and Gamma. Animals vaccinated with 10^8^ vp Ad26.COV2.S also regained activity faster after challenge with SARS-CoV-2 B.1.22 and Delta than mock-vaccinated animals, which was reflected by a higher AUC for activity change after SARS-CoV-2 B.1.22 infection. However, no significant protection against activity loss was observed in hamsters vaccinated with 10^8^ vp Ad26.COV2.S after SARS-CoV-2 Gamma challenge. Moreover, we found that weight change partially correlates with activity change, which supports using activity change as a complementary readout for vaccine efficacy (Supplemental Fig. 2).

**Fig. 2:**
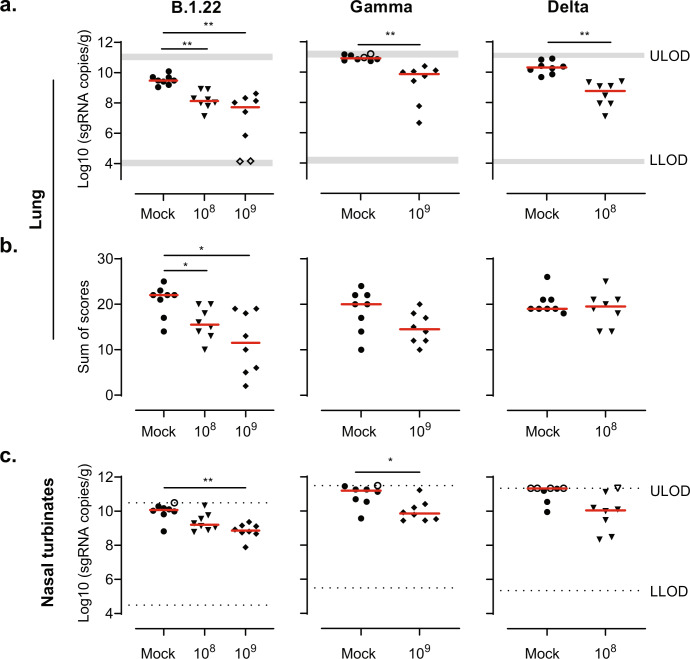
Ad26.COV2.S reduces the viral load and lung histopathology after challenge with SARS-CoV-2 B.1.22, Gamma and Delta. Hamsters were vaccinated with formulation buffer (mock), 10^8^ or 10^9^ vp Ad26.COV2.S at day −28 (*n* = 8 per group). The animals were intranasally challenged with 10^3^ TCID_50_ SARS-CoV-2 B.1.22, 10^4^ TCID_50_ SARS-CoV-2 Gamma, or 10^4^ TCID_50_ SARS-CoV-2 Delta on day 0. While animals vaccinated with 10^8^ and 10^9^ vp were evaluated on day 4 after the challenge with SARS-CoV-2 B.1.22, only animals vaccinated with 10^9^ vp were examined on day 4 after the challenge with SARS-CoV-2 Gamma, and only 10^8^ vp-vaccinated animals were evaluated at day 4 after challenge with the Delta variant. SARS-CoV-2 Envelope subgenomic RNA (sgRNA) was measured in the **a** lungs and **c** nasal turbinates (nose) on day 4. **b** Paraffin sections from lung tissue (H&E) were scored at day 4 for alveolar edema, hemorrhage, infiltrate, alveolar and/or interstitial inflammation, bronchitis and/or bronchiolitis, hyperplasia and/or hypertrophy mucous cells, peribronchiolar and/or perivascular cuffing, pleural fibrosis, thickening of alveolar septa and type II pneumocyte hyperplasia. The sum of scores is presented (potential range 0–48). Red horizontal bars indicate the median response per group and the dotted line/gray zone indicates the limit of detection (LOD) or LOD range. Open symbols indicate the response is at or below the lower LOD (LLOD)/at or above the upper LOD (ULOD). Comparisons were performed by a Mann–Whitney *U* test with a twofold Bonferroni correction. Statistical differences are indicated by asterisks: *P < 0.05, **P < 0.01.

Lung and nasal turbinate tissues were collected 4 days after infection from a separate cohort to quantify the viral replication and assess the lung histopathology. While animals vaccinated with 10^8^ and 10^9^ vp Ad26.COV2.S were evaluated after challenge with SARS-CoV-2 B.1.22, animals vaccinated with 10^9^ vp were examined after challenge with SARS-CoV-2 Gamma, and animals vaccinated with 10^8^ vp were evaluated after challenge with the Delta variant. SARS-CoV-2 Envelope (E) subgenomic Envelope RNA (sgRNA) was measured by qPCR to quantify viral replication in the lungs and nasal turbinates. Animals vaccinated with Ad26.COV2.S had significantly lower sgRNA levels in the lungs after challenge with all three variants, compared with the mock-vaccinated group (Fig. 2a). Correspondingly, the number of SARS-CoV-2 nucleocapsid-positive pneumocytes was reduced in the lung parenchyma and bronchi/bronchioles of vaccinated animals after SARS-CoV-2 B.1.22, Gamma and Delta challenge (Supplemental Fig. 3). Moreover, vaccination with either 10^8^ or 10^9^ vp Ad26.COV2.S reduced signs of lower respiratory tract histopathology after challenge with SARS-CoV-2 B.1.22, while histopathology after Gamma challenge with 10^9^ vp Ad26.COV2.S and Delta with 10^8^ vp Ad26.COV2.S was reduced but did not reach statistical significance (Fig. 2b). In addition, we also observed protection against viral replication by Ad26.COV2.S in the nasal turbinates after challenge with either SARS-CoV-2 B.1.22 and Gamma, while protection against the Delta variant did not reach statistical significance (Fig. 2c).

**Fig. 3:**
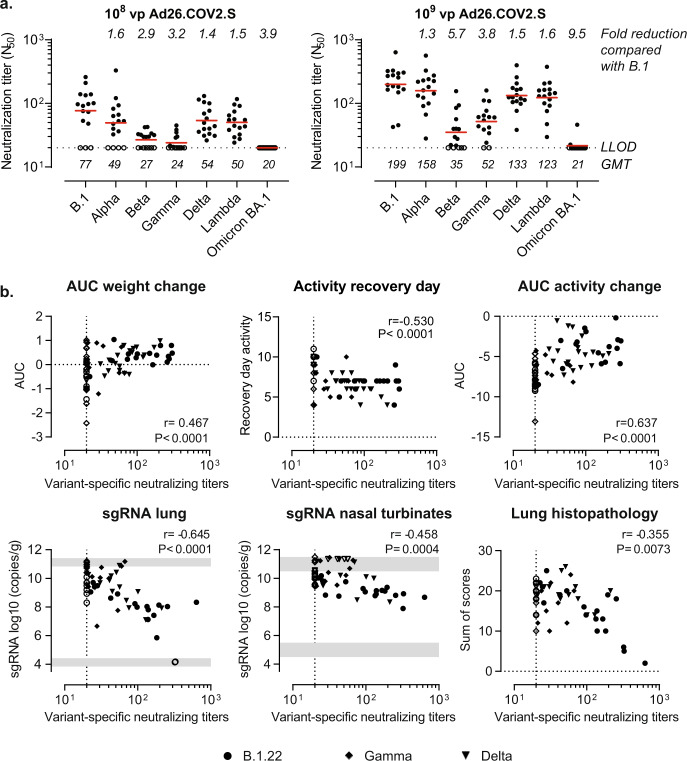
Correlation of vaccine efficacy parameters with neutralizing antibody titers. Hamsters were vaccinated with formulation buffer (mock), 10^8^ or 10^9^ vp Ad26.COV2.S at day −28. Sera were collected before the challenge at day 0 to measure neutralizing titers against pseudotyped viruses expressing SARS-CoV-2 spike protein variants in a pseudotyped virus neutralization assay (psVNA). Neutralizing antibody titers are expressed as the dilution giving a 50% reduction (N_50_) in the normalized luciferase readout. **a** Neutralizing antibody titers are shown for various spike protein variants. **b** Hamsters were intranasally challenged with 10^3^ TCID_50_ SARS-CoV-2 B.1.22, 10^4^ TCID_50_ SARS-CoV-2 Gamma, or 10^4^ TCID_50_ SARS-CoV-2 Delta on day 0. The correlation between neutralizing antibody titers homologous to the challenge strain (B.1 nAbs for B.1.22 challenge, P.1 nAbs for Gamma challenge, and B.1.617.2 nAbs for Delta challenge) in animals vaccinated with Ad26.COV2.S or formulation buffer (mock) with weight change, activity change (recovery day and AUC), lung histopathology, and SARS-CoV-2 Envelope subgenomic RNA (sgRNA) viral load in the lungs and nasal turbinates is shown. Correlation coefficients were calculated across all 3 VOC using a two-sided Spearman rank correlation. Red horizontal bars indicate the geometric mean titers (GMT) and the dotted line/gray zone indicates the limit of detection (LOD) or LOD range. Open symbols indicate the response is at or below the lower LOD (LLOD)/at or above the upper LOD (ULOD).

We also evaluated the breadth of neutralization by a pseudotyped virus neutralizing assay (psVNA) 4 weeks after vaccination with Ad26.COV2.S. While neutralizing antibody titers to Alpha, Delta, and Lambda spikes were 1.3- to 1.6-fold reduced relative to B.1, we observed a 2.9- to 5.7-fold reduction against Beta and Gamma spike (Fig. 3a). Nevertheless, the protective efficacy against weight loss (Fig. 1), activity loss (Supplemental Fig. 1), viral load and lung histopathology (Fig. 2) was largely maintained. Neutralizing antibody titers homologous to the challenge strain partially correlated with weight change, activity change, lung viral load, and lung histopathology (Fig. 3b). However, Omicron BA.1 neutralizing antibody were largely undetectable (Fig. 3a) which is in line with the extensive evasion of neutralizing responses by previous infections and vaccination in humans^6,7^.

### Ad26.COV2.S.529 is immunogenic in naive mice

To assess whether the neutralizing antibody titers against the Omicron variant could be augmented, we generated the Omicron BA.1-based vaccine candidate Ad26.COV2.S.529. Spike expression and antigenicity by Ad26.COV2.S.529 were characterized in vitro and compared with Ad26.COV2.S. Spike protein expression was evaluated after transduction of A549 cells using a quantitative cell-based ELISA, which indicated that spike expression and antigenicity after transduction with Ad26.COV2.S or Ad26.COV2.S.529 were comparable (Supplemental Fig. 4).

**Fig. 4:**
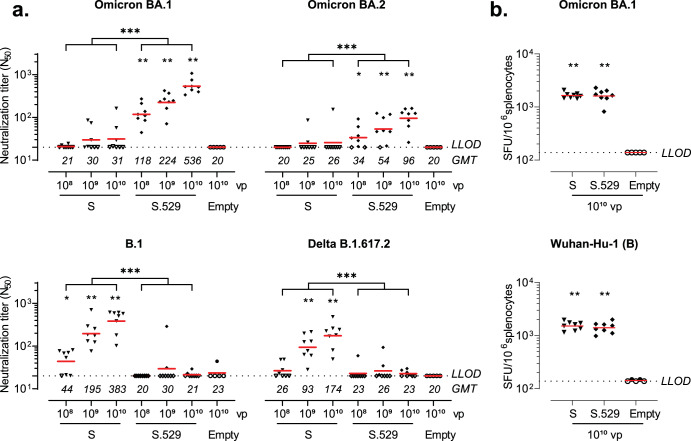
Neutralizing antibodies and cellular responses induced by Ad26.COV2.S.529 in naive mice. Mice were vaccinated with 10^8^, 10^9^, 10^10^ viral particles (vp) Ad26.COV2.S (S) or Ad26.COV2.S.529 (S.529, *n* = 8 per dose); 10^10^ vp of the negative control vector Ad26.Empty (Empty, *n* = 5) at day 0. **a** Serum was sampled 6 weeks after vaccination to measure neutralizing titers against pseudotyped viruses expressing SARS-CoV-2 B.1, Delta B.1617.2, Omicron BA.1 or BA.2 spike in a pseudotyped virus neutralization assay (psVNA). Neutralizing antibody titers are expressed as the dilution giving a 50% reduction (N_50_) in the normalized luciferase readout. **b** Spike protein–specific IFN-γ T cell responses as measured in splenocytes collected 6 weeks after vaccination with 10^10^ vp S, S.529 or Empty. Spot-forming units (SFU) per 1 × 10^6^ splenocytes are shown. Horizontal red bars per vaccination group represent geometric mean titers (GMT). Dashed horizontal lines represent the lower limit of detection (LLOD). Open symbols indicate the response was at or below LLOD. Comparisons to the mock group were performed by a Mann–Whitney test, comparisons between Ad26.COV2.S and Ad26.COV2.S.529 were performed by a Cochran–Mantel–Haenszel test across dose or a *t* test. Significance is shown compared with the mock group, unless specified otherwise. Statistical differences are indicated by asterisks: *P < 0.05, **P < 0.01, ***P < 0.001.

Naive mice vaccinated with 10^8^, 10^9,^ or 10^10^ vp Ad26.COV2.S.529 elicited dose-dependent Omicron BA.1 spike neutralizing antibody titers 6 weeks after vaccination as measured by psVNA, while BA.1 neutralizing antibodies were largely undetectable after vaccination with Ad26.COV2.S (Fig. 4a). Serum from Ad26.COV2.S.529-vaccinated mice also neutralized Omicron BA.2, but neutralizing antibody titers were 3.5- to 5.5-fold lower compared with Omicron BA.1. Conversely, Ad26.COV2.S induced dose-dependent B.1 (D614G) and Delta B.1.617.2 spike neutralizing responses 6 weeks after vaccination, while neutralizing titers were largely undetectable in the Ad26.COV2.S.529- and Ad26.Empty (mock)-vaccinated groups (Fig. 4a).

Spike protein–specific IFN-γ T cell responses were also measured 6 weeks after vaccination with 10^10^ vp Ad26.COV2.S or Ad26.COV2.S.529 by ELISpot using splenocytes stimulated with 15-mer peptides overlapping by 11 amino acids and spanning the complete SARS-CoV-2 Wuhan-Hu-1 (B) or Omicron BA.1 spike protein. IFN-γ responses after stimulation with SARS-CoV-2 Wuhan-Hu-1 (B) or Omicron BA.1 spike protein peptide pools were comparable in the groups that received Ad26.COV2.S and Ad26.COV2.S.529 (Fig. 4b).

### Ad26.COV2.S.529 is immunogenic in pre-immune hamsters

**Fig. 5:**
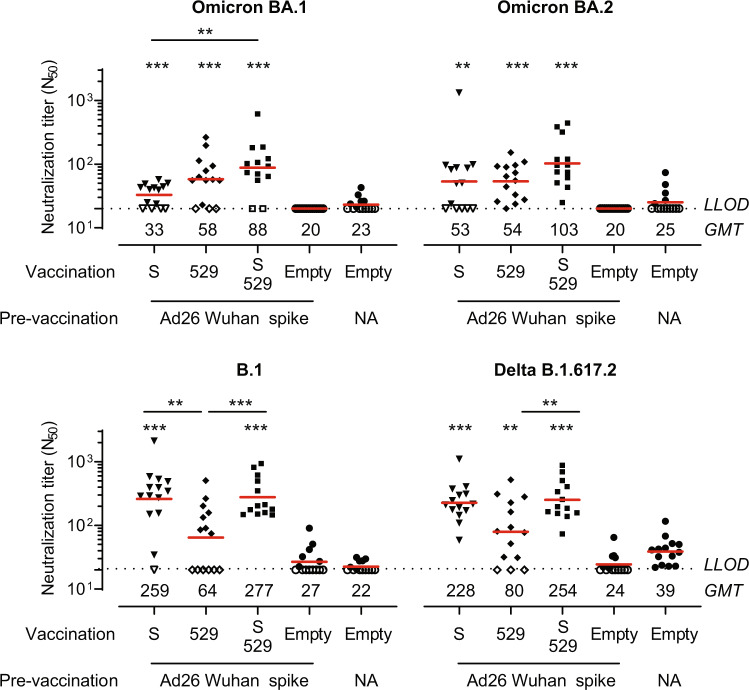
SARS-CoV-2 spike variant neutralizing antibodies induced by Ad26.COV2.S.529 in pre-immune hamsters 23 weeks after pre-exposure. Hamsters with pre-existing immunity were generated by pre-vaccination with 10^7^ viral particles (vp) of Ad26NCOV006 (Ad26 vector encoding Wuhan-Hu-1 spike, pre-immune animals) at week −27. Naive control hamsters were not vaccinated (NA). At week −4, Ad26 Wuhan-Hu-1 spike-vaccinated hamsters were vaccinated with 10^9^ vp Ad26.COV2.S (S), Ad26.COV2.S.529 (529), a 1:1 combination of S and 529 or Ad26.Empty (all *n* = 13–14). In addition, naive hamsters were vaccinated with Ad26.Empty (*n* = 14). Serum was collected at week 0 for psVNA analysis. Neutralizing antibody titers are expressed as the dilution giving a 50% reduction (N_50_) in the normalized luciferase readout (normalization relative to control wells without any serum added). Horizontal red bars and values per vaccination group represent geometric mean (GMT) titers. Dashed horizontal lines represent the lower limit of detection (LLOD). Open symbols indicate the response is at or below the LLOD. Comparisons to the pre-vaccinated Ad26.Empty group and pairwise comparisons were performed by a *t* test, Tobit *Z* test, or Mann–Whitney test with a threefold Bonferroni correction. Significance is shown compared with the pre-vaccinated-only group unless depicted otherwise. Statistical differences are indicated by asterisks: **P < 0.01, ***P < 0.001.

As an increasing part of the population acquired pre-existing immunity either by infection or vaccination^18,19^, we also evaluated the immunogenicity of Ad26.COV2.S.529 in hamsters with pre-existing immunity to a Wuhan-Hu-1 SARS-CoV-2 spike protein. Hamsters were first pre-vaccinated with a low dose (10^7^ vp) of Ad26NCOV006, which encodes the non-stabilized ancestral SARS-CoV-2 Wuhan-Hu-1 spike protein, to induce pre-existing immunity at a level that does not provide protection against SARS-CoV-2 B.1 challenge^15^. After ~5.5 months, the hamsters received a vaccination with 10^9^ vp Ad26.COV2.S, Ad26.COV2.S.529, a 1:1 combination of 5 × 10^8^ vp Ad26.COV2.S and 5 × 10^8^ vp Ad26.COV2.S.529, or 10^9^ vp of the negative control vector Ad26.Empty. Neutralization titers against spike variants were evaluated using a psVNA assay in sera collected 4 weeks after vaccination. In contrast to what we observed in naive mice, Ad26.COV2.S and Ad26.COV2.S.529 induced comparable Omicron BA.1 and BA.2-neutralizing titers in pre-immune hamsters (Fig. 5). Omicron BA.1 neutralizing titers were higher in animals vaccinated with a combination of Ad26.COV2.S and Ad26.COV2.S.529 compared with Ad26.COV2.S, but Omicron BA.2-neutralizing titers were not significantly higher in animals vaccinated with the combination vaccine compared with Ad26.COV2.S or Ad26.COV2.S.529. Ad26.COV2.S.529 induced significantly lower B.1 neutralizing antibody titers compared with both Ad26.COV2.S and the combination vaccine, while Delta neutralizing titers were significantly lower in the Ad26.COV2.S.529 group compared with the combination vaccine group. However, both B.1 and Delta B.1.617.2-neutralizing antibody titers were comparable in pre-immune hamsters that received Ad26.COV2.S and the combination vaccine.

### Ad26.COV2.S and Ad26.COV2.S.529 provide comparable protection against SARS-CoV-2 Omicron BA.2 in hamsters

Next, the same animals were challenged with SARS-CoV-2 Omicron BA.2 to evaluate the efficacy of Ad26.COV2.S, Ad26.COV2.S.529 and a 1:1 combination of the two vaccines. In contrast to the SARS-CoV-2 B.1.22, Gamma and Delta variants, BA.2 challenge resulted in minor weight- and activity loss in mock-vaccinated hamsters (Supplemental Fig. 5) as also observed by others^20,21^. Hence, in this study weight and activity loss were not used as a measure of vaccine protection.

**Fig. 6:**
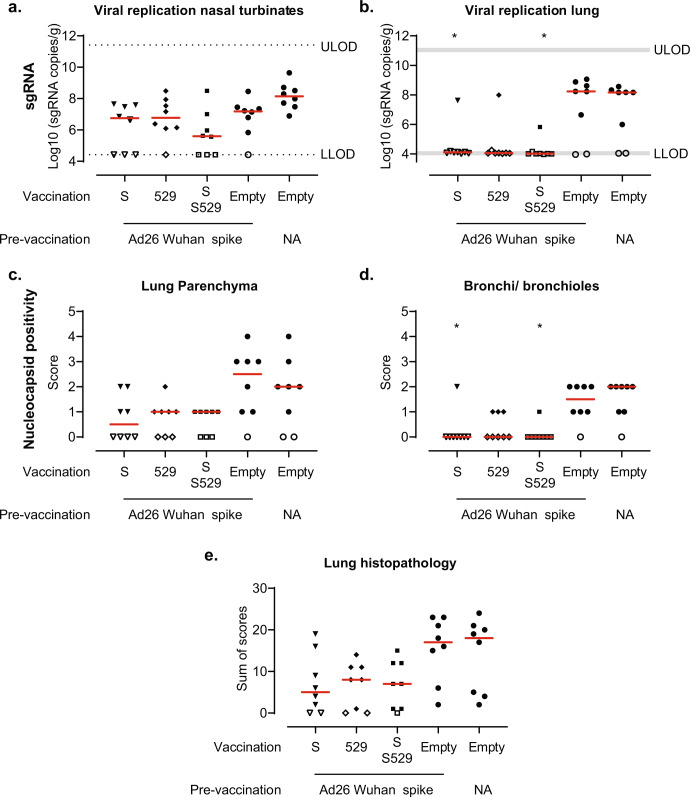
Protection against the SARS-CoV-2 BA.2 variant. Hamsters with pre-existing immunity were generated by pre-vaccination with 10^7^ viral particles (vp) of Ad26NCOV006 (Ad26 vector encoding Wuhan-Hu-1 spike, pre-immune animals) at week −27. Naive control hamsters were not vaccinated (NA). At week −4, Ad26 Wuhan-Hu-1 spike-vaccinated hamsters were vaccinated with 10^9^ vp Ad26.COV2.S (S), Ad26.COV2.S.529 (529), a 1:1 combination of S and 529 or Ad26.Empty (all *n* = 8). In addition, naive hamsters were vaccinated with Ad26.Empty (*n* = 8). The animals were intranasally challenged with 10^4.7^ TCID_50_ SARS-CoV-2 BA.2 in week 0. SARS-CoV-2 Envelope subgenomic RNA (sgRNA) viral load was measured in the **a** nasal turbinates and **b** lungs. **c**/**d** Lungs (parenchyma and bronchi/bronchioles) were stained and scored for SARS-CoV-2 nucleocapsid-protein positivity (score 0–5). **e** Paraffin sections from lung tissue (H&E) were scored on day 4 for lower tract histopathology. The sum of scores is presented (maximum possible score of 48). Red horizontal bars indicate the median response per group and the dotted line/gray zone indicates the limit of detection (LOD) or LOD range. Open symbols indicate the response is at or below the lower LOD (LLOD)/at or above upper LOD (ULOD) or a score of 0 for nucleocapsid positivity or histopathology. Comparisons to the pre-vaccination-only group and pairwise comparisons were performed by a Mann–Whitney *U* test with a threefold Bonferroni correction. Significance is shown compared with the pre-vaccinated-only group unless depicted otherwise. Statistical differences are indicated by asterisks: *P < 0.05.

Lung and nasal turbinate tissues were collected 4 days after infection from a separate cohort to quantify viral replication and assess lung histopathology. In contrast to the reduced viral replication in the nasal turbinates in vaccinated animals after SARS-CoV-2 B.1.22, Gamma, and Delta challenge, we did not observe statistically significant protection against viral replication in the nasal turbinates by Omicron BA.2 compared with the pre-vaccination-only group (Fig. 6a). While we detected viral replication in the lungs of 6 out of 8 naive or pre-vaccinated-only control hamsters, sgRNA in the lung was undetectable in the lungs in seven out of eight hamsters after vaccination with Ad26.COV2.S, Ad26.COV2.S.529 and the combination (Fig. 6b). In line with these results, we observed a trend towards reduced or non-detectable viral replication in the lung parenchyma which was significantly reduced in the bronchi/bronchioles in animals vaccinated with Ad26.COV2.S or a combination of Ad26.COV2.S and Ad26.COV2.S.529 (Fig. 6c, d), compared with the pre-vaccinated-only group. We also observed a trend towards reduced signs of lower respiratory tract histopathology in vaccinated animals (Fig. 6e, Supplemental Fig. 6). Overall, we observed comparable protection by Ad26.COV2.S, Ad26.COV2.S.529 and the combination against Omicron BA.2 challenge in pre-vaccinated hamsters.

## Discussion

In response to the COVID-19 outbreak, Janssen developed the Ad26.COV2.S vaccine encoding a pre-fusion conformation stabilized SARS-CoV-2 spike protein based on the Wuhan-Hu-1 isolate. Several rapidly spreading SARS-CoV-2 VOC such as the Gamma, Delta, and Omicron variants have emerged since the outbreak of the pandemic that carry mutations in the spike protein that enables partial evasion of immunity induced by vaccination and infection. Here, we showed that the protection against the Gamma and Delta variant by Ad26.COV2.S was maintained in hamsters, supportive of the largely maintained efficacy against severe disease in humans during surges of infections caused by SARS-CoV-2 VOC^3,22,23^. This suggests that hamster efficacy studies may be employed to predict the vaccine efficacy against (emerging) VOC in humans.

While a booster dose with Wuhan-Hu-1 spike-based mRNA vaccines or Ad26.COV2.S also provides strong protection against Omicron infection-related severe disease in humans^8^, protection from mild and asymptomatic disease is lower^24^ and decreases faster^25^. Here, we evaluated the immunogenicity and efficacy of boosting with Ad26.COV2.S, an Omicron BA.1-based vaccine candidate (Ad26.COV2.S.529), and a combination of the two vaccines against Omicron BA.2 in pre-immunized hamsters. In contrast to challenge of hamsters with the SARS-CoV-2 variants B.1.22, Gamma, and Delta, challenge of mock-vaccinated hamsters with Omicron BA.2 did not induce weight loss in line with previous studies with BA.2^20,21^ and BA.1^26–34^. We refined the hamster model by also measuring activity change as an indicator of clinical health, which partially correlated with weight change after SARS-CoV-2 B.1.22, Gamma and Delta challenge. While we did observe activity loss in mock-vaccinated hamsters after Omicron BA.2 challenge, this was much less severe than after challenge with ancestral variants. However, viral replication and pneumonia were detected after Omicron BA.2 challenge.

Vaccination with Ad26.COV2.S, Ad26.COV2.S.529 and a combination of the two vaccines provided strong and comparable protection against Omicron BA.2 challenge in hamsters with pre-existing immunity to SARS-CoV-2 Wuhan-Hu-1 spike. Omicron BA.1 mRNA- and Ad26-based booster vaccines also provided equivalent or only slightly higher protection against Omicron BA.1 challenge in Wuhan-Hu-1 spike-vaccinated NHPs than ancestral Wuhan-Hu-1-based booster vaccines^35,36^. In line with the equivalent protection observed against Omicron BA.2, Omicron BA.2-neutralizing titers were comparable after vaccination with Ad26.COV2.S, Ad26.COV2.S.529 and a combination of the two vaccines in hamsters with pre-existing immunity to Wuhan-Hu-1 spike. A booster with Omicron BA.1 or BA.4/5-based vaccines also elicits similar or maximally twofold higher Omicron BA.1 or BA.4/5 neutralizing titers compared with ancestral-based vaccines in humans^37–39^, NHPs^35,36^ and mice^40–42^ that were previously vaccinated with ancestral-based vaccines. This suggests that Omicron-based vaccines predominantly boost pre-existing immunity against shared epitopes instead of generating *de novo* responses in a population that received ancestral spike-based vaccines^43^, which has also been shown for Omicron BA.1, BA.2 and BA.5 breakthrough infection^44–48^. However, Omicron-specific B-cells have been shown to emerge later (4–6 months, although still at a low frequency) after Omicron BA.1 breakthrough infection, which may be attributed to *de novo* responses or affinity maturation of pre-existing cross-reactive memory B-cells towards Omicron^49^. Therefore, Omicron-based boosters may provide a protective advantage after a longer follow-up than the 1-month interval between boost and challenge in this study. A second Omicron-based booster or Omicron breakthrough infection has the potential to expand these Omicron-specific responses and further improve protection^50^. In addition, Omicron (BA.4/BA.5)-based booster vaccines may be more immunogenic than ancestral-based boosters against newer Omicron variants, such as BQ.1.1^51^.

In contrast to hamsters with pre-existing immunity, Omicron BA.1 and BA.2-neutralizing antibody titers were higher after vaccination with Ad26.COV2.S.529 than with Ad26.COV2.S in naive mice as previously reported with Omicron RNA and Ad26 vaccines^36,40–42,52–55^. Therefore, vaccination with Omicron BA.1-based vaccines may improve protection against circulating Omicron variants in an immunologically naive population, as has been shown with Ad26.COV2.S.529 in naive NHPs^36^. However, the naive population continues to decrease as the majority of the population now acquired pre-existing immunity by vaccination or infection^18,19^. Although Omicron-based vaccines may improve protection in an immunologically naive population, our data in hamsters with pre-existing immunity to Wuhan-Hu-1 spike suggests that a Wuhan-Hu-1- and an Omicron BA.1-based booster vaccine confer comparable strong protection to Omicron BA.2. This data supports real-world evidence^8,56^ vaccine-effectiveness studies showing that boosting with Wuhan spike-based vaccines provides robust protection from severe COVID-19 disease due to Omicron infection.

## Methods

### Vaccines and challenge stocks

Replication-incompetent E1/E3-deleted adenovirus serotype 26 (Ad26) vector-based vaccines were generated using the AdVac system as described previously^2^. Ad26NCOV006 and Ad26.COV2.S encode a SARS-CoV-2 spike protein sequence based on the SARS-CoV-2 Wuhan-Hu-1 spike protein (GenBank accession number MN908947), while Ad26.COV2.S.529 encodes a SARS-CoV-2 spike protein sequence based on the SARS-CoV-2 Omicron BA.1 spike protein (GISAID accession number EPI_ISL_6913991). Spike proteins encoded by Ad26.COV2.S and Ad26.COV2.S.529 were stabilized in the pre-fusion confirmation by the proline substitutions K986P and V987P and substitutions R682S and R685G, which abolish the furin cleavage site. Ad26.Empty, which did not contain a transgene, or formulation buffer was used as a negative control.

The SARS-CoV-2 Gamma isolate hCoV-19/Japan/TY7-503/2021 (NR-54982; P.1) was obtained from Biodefense and Emerging Infections Research Resources Repository (BEI Resources). SARS-CoV-2 B.1.22^17^, Delta AY.5, and Omicron BA.2 were isolated from nasal and/or oropharyngeal swabs from human subjects by Wageningen Bioveterinary Research (WBVR). Virus challenge stocks were prepared as described previously^17^ using one or two low-MOI passages in Vero E6, Vero-TMPRSS2, or Calu-3 cells. Deep sequencing of these stocks confirmed the presence of the key lineage mutations or deletions in Spike in Supplemental Table 1 compared with the Wuhan-Hu-1 isolate (MN908947; lineage B), while no additional mutations/deletions in the spike protein gene were found with a frequency >1%.

### Cell-based ELISA

A549 cells were seeded at 2.9 × 10^4^ cells/well in Dulbecco’s Modified Eagle Medium (DMEM) with 10% heat-inactivated fetal bovine serum (FBS) in a flat-bottomed 96-well microtiter plate (Corning). The plate was incubated overnight at 37 °C in 10% CO_2_. After 24 hours, cells were transduced with either Ad26.COV2.S or Ad26.COV2.S.529 at a dose of 2000 infectious units [IU]/cell and the cells were incubated for 48 hours at 37 °C in 10% CO_2_. Two days post transduction, cells were washed four times with phosphate-buffered saline (PBS) and subsequently fixed with 4% formaldehyde in PBS. After a 20-minute incubation at room temperature (RT), cells were washed four times with 0.05% Tween-20 in PBS. Cells were permeabilized by incubation with 1% Elugent (Merck) for 15–20 minutes, after which they were washed four times with 0.05% Tween-20 in PBS. Casein blocking buffer (Thermo Scientific) was then added and the cells were incubated for 60 to 90 minutes at 37 °C in 10% CO_2_. Cells were washed four times with 0.05% Tween-20 in PBS, after which twofold diluted CV3–25 antibody or ACE2-Fc fusion protein (0.007–15 µg/ml) was added per well. CV3–25 was produced at ImmunoPrecise according to Jennewein et al.^57^ and ACE2-Fc was prepared according to Liu et al.^58^. After 30 to 60 minutes of incubation, cells were washed four times with 0.05% Tween-20 in PBS. Next, cells were incubated with mouse HRP-conjugated anti-human IgG Fc (Jackson Immunoresearch, cat 209-035-098, 1:8000) for 40 minutes at RT. The cells were washed four times with 0.05% Tween-20 in PBS. 3,3′,5,5′-Tetramethylbenzidine (TMB) Liquid Substrate System for ELISA (Sigma) was added and after 20 minutes the reaction was terminated by adding Stop Reagent for TMB Substrate (Sigma). Signal was measured at 450 nm (signal) and at 630 nm (for background subtraction) using a Biotek Synergy Neo reader. Data were analyzed using GraphPad Prism 9, using the “Specific binding with Hill slope” module to calculate the antibody binding affinity.

### Animal studies

Animal experiments were approved by the Central Authority for Scientific Procedures on Animals (Centrale Commissie Dierproeven), the institutional animal welfare body, and conducted in accordance with the European guidelines (EU directive on animal testing 2010/63/EU and ETS 123) and local Dutch legislation on animal experiments. Female BALB/c mice aged 8–10 weeks at the start of the study were purchased from Charles River Laboratories (Germany). Mice were vaccinated intramuscularly with 100 μl vaccine under general anesthesia with isoflurane. At the end of the study, the animals were exsanguinated by cardiac puncture under isoflurane anesthesia and sacrificed by cervical dislocation after which spleens were collected.

Female Syrian golden RjHan:AURA hamsters, aged 9–14 weeks at the start of the study, were purchased from Janvier Labs (France). Hamsters were vaccinated intramuscularly with 100 μl vaccine under general anesthesia with isoflurane or under general anesthesia with medetomidine and ketamine applied intraperitoneally and antagonized with atipamezole applied subcutaneously after the procedure. Blood samples were collected via the retro-orbital route under anesthesia as described above. The in-life phase of hamster challenge studies was performed at Wageningen Bioveterinary Research (WBVR), Lelystad, the Netherlands. Hamsters were intranasally inoculated with 100 µl (50 µL per nostril) containing 10^3^ TCID_50_ SARS-CoV-2 B.1.22, 10^4^ TCID_50_ SARS-CoV-2 Gamma P.1, 10^4^ TCID_50_ SARS-CoV-2 Delta AY.5 or 10^4.7^ TCID_50_ SARS-CoV-2 Omicron BA.2 under general anesthesia with medetomidine and ketamine applied intraperitoneally and antagonized with atipamezole, applied subcutaneously after the procedure. The activity of the animals was monitored using activity wheels (Tecnilab-BMI, 2154F0105). At the end of the experiment, hamsters were exsanguinated via the aorta and vena cava following intraperitoneal (i.p.) injection of medetomidine/ketamine and necropsy was performed. Lung and nasal turbinate tissues were collected to quantify the viral replication and assess the lung histopathology. Blood from all animal experiments was processed for serum isolation.

### Viral load

Right lungs were weighed and homogenized in 5 ml PBS using an Ultra-Turrax homogenator with BMT-20M-S tubes with stainless steel balls (both IKA). Right nasal turbinates were weighed and homogenized in 1 ml Eagle’s Minimum Essential Medium (EMEM) supplemented with 1% antibiotic-antimycotic, 1% sodium bicarbonate, 1% L-glutamine and 1% nonessential amino acids (all Life Technologies) in Lysing Matrix tubes with a Fastprep-24 Classic instrument (both MP Biomedicals). RNA was extracted from lung or nasal turbinate homogenates using the High Pure RNA Isolation kit according to the manufacturer’s instructions (Roche). SARS-CoV-2 Envelope (E) subgenomic RNA (sgRNA) was quantified using the SuperScript III One-Step RT-PCR System. The reactions were carried out in a volume of 25 µl with 4 µl RNA, the Platinum Taq DNA Polymerase (Invitrogen), and the primers/probes as published by Wölfel^59^ and Corman^60^. Reverse transcription was performed at 50 °C for 15 minutes, followed by enzyme activation at 95 °C for 2 minutes and 40 PCR cycles of 95 °C for 15 seconds and 60 °C for 30 seconds.

RNA standards were prepared from a pcDNA3.1 plasmid containing the complete E gene behind the SARS-CoV-2 subgenomic leader sequence (nucleotides 1–77 of the SARS-CoV-2 genome). Serial dilution of sgRNA standard with a known number of copies was taken along to calculate sgRNA copies. The number of copies is expressed as log10 copies/gram tissue.

### Histopathology and immunohistochemistry

Left lung lobes were fixed in 10% neutral-buffered formalin, routinely processed, paraffin-embedded, micro-sectioned to 5 µm on glass slides, and stained with haematoxylin and eosin (H&E) using the Ventana HE 600 system (Roche) for histopathological evaluation. The H&E-stained tissue sections were examined by light microscopy and scored from 0–5 for alveolar infiltrate, alveolar hemorrhage, alveolar edema, thickening of alveolar septa, type II pneumocyte hyperplasia, bronchitis and/or bronchiolitis, pleural fibrosis, mucous cell hyperplasia and/or hypertrophy: 0 (no change), 1 (minimal histological change), 2 (slight/mild), 3 (moderate), 4 (marked) and, 5 (severe/massive histopathological change). Pleural fibrosis and hyperplasia/hypertrophy of mucous cells are considered to be regenerative findings and were not observed at day 4 post infection. The extent of alveolar and/or interstitial inflammation was also scored from 0–5, where: 0 (none), 1 (focal to multifocal, < 10% of tissue), 2 (multifocal, 10–35% of tissue), 3 (multifocal or multifocal to coalescing, 35–65% of tissue), 4 (multifocal to diffuse, >65% of tissue), and 5 (diffuse, 100% of tissue). Peribronchiolar and/or perivascular cuffing was scored from 0–3: 0 (0 cells thick), 1 (1–2 cells thick), 2 (3–10 cells thick), 3 (>10 cells thick). The cumulative score for the extent and severity of inflammation of lung tissues was defined as the sum of the above parameters, adding up to a possible maximum score of 48.

Paraffin sections of lungs of all animals were immunohistochemically stained with the polyclonal rabbit SARS-CoV/SARS-CoV-2 Nucleocapsid Antibody (SinoBiological 40143-T62, dilution 1/6000). The immunohistochemically stained tissue sections were examined by light microscopy and the number of SARS-CoV-2 nucleocapsid-protein (N)-positive cells were scored in lung parenchyma and in bronchi/bronchioles: 0 (none), 1 (minimal), 2 (mild), 3 (moderate), 4 (marked) and, 5 (massive).

### Recombinant lentivirus-based pseudotyped virus neutralization assay (psVNA)

Neutralizing antibody titers were measured against several SARS-CoV-2 spike variants by a pseudotyped virus neutralization assay (psVNA). Human Immunodeficiency Virus (HIV)-based lentiviruses, pseudotyped with SARS-CoV-2 spike protein (based on Wuhan‐Hu‐1; GenBank accession number MN908947) were generated as described previously^61,62^. Substitutions and deletions in the spike protein open reading frame for the variant B.1, B.1.1.7 (Alpha), B.1.351 (Beta), P.1 (Gamma), B.1.617.2/AY.5 (Delta), C.37 (Lambda), BA.1, BA.2 (both Omicron) were introduced using standard molecular biology techniques and confirmed by sequencing (Supplemental Table 1).

Assays were performed on HEK293T target cells stably expressing the human angiotensin-converting enzyme 2 (ACE2) and human transmembrane serine protease 2 (TMPRSS2) genes (VectorBuilder). The cells were seeded in white half-area 96-well tissue culture plates (Perkin Elmer) at a density of 1.5 × 10^4^ cells/well. Twofold serial dilutions were prepared from heat-inactivated serum samples in phenol red-free DMEM supplemented with 1% FBS and 1% Penicillin/Streptomycin. The serially diluted serum samples were incubated at room temperature with an equal volume of pseudoviral particles with titers of approximately 1 × 10^5^ Relative Luminescence Units (RLU) luciferase activity. After one hour incubation, the serum-particle mixture was inoculated onto HEK293T.ACE2.TMPRSS2 cells. Luciferase activity was measured 40 hours after transduction by adding an equal volume of NeoLite substrate (Perkin Elmer) to the wells according to the manufacturer’s protocol, followed by readout of RLU on the EnSight Multimode Plate Reader (Perkin Elmer). SARS-CoV-2-neutralizing titers were calculated using a four-parameter curve fit as the sample dilution at which a 50% reduction (N_50_) of luciferase readout was observed compared with luciferase readout in the absence of serum (High Control). The starting serum sample dilution of 20 was fixed as the lower limit of detection (LLOD).

### IFN-γ enzyme-linked immune-spot assay (ELISpot)

An IFN-γ ELIspot was performed on freshly isolated splenocytes using the mouse IFN-γ ELISpot PLUS kit ALP (Mabtech). Spleens were isolated and processed into single-cell suspensions using the gentleMACS™ Dissociator system (Miltenyi Biotec) after which red blood cells were lysed using ACK lysis buffer (Lonza). Cells were plated in pre-coated plates at a concentration of 1 × 10^5^ cells per well and stimulated with either cell culture medium in the presence of DMSO, two Wuhan-Hu-1 (B) spike pools, or Omicron BA.1 spike pools (PM-SARS2-SMUT08–1) or 50 ng/ml PMA and 2 µg/ml ionomycin as positive control for 18 hours. Spike pools consisted of consecutive 15-mer peptides with 11 amino acid overlap (JPT) spanning the entire length of the SARS-CoV-2 spike protein at a peptide concentration of 1 µg/ml. Analysis was performed using the Eli.Expter reader and Eli.Analyse V6.1 (both A.EL.VIS). Spot-forming units (SFU) per 1 × 10^6^ splenocytes were calculated per pool and summed across the two peptide pools per variant. The LLOD was based on the 95th percentile of the background response in the wells stimulated with cell culture medium in the presence of DMSO.

### Statistical analysis

Statistical comparisons were performed in SAS 9.4 using ANOVA with a posthoc *t* test. If titers were censored at LLOD, then a Tobit z-test from a Tobit ANOVA was used instead. If a vaccine-dose had more than 50% censored measurements, the non-parametric Mann–Whitney *U* test or Cochran–Mantel–Haenszel test for across-dose comparisons was used instead. The area under the curve (AUC) of the relative bodyweight change and activity (both as fraction * day) compared with the average pre-challenge weight/activity was calculated with the trapezoidal rule. Missing bodyweight and activity datapoints were filled in using the last observation carried forward (LOCF) method. Correlation coefficients were calculated using two-sided Spearman rank correlation in GraphPad Prism 9.4.1. For all statistical tests the significance level was 5%. A Bonferroni correction was applied to adjust for multiple comparisons.

### Reporting summary

Further information on research design is available in the Nature Research Reporting Summary linked to this article.

## Supplementary information

Supplemental figures

REPORTING SUMMARY

## Data Availability

All data that support the findings of this study are available from the corresponding author upon request.
