# Supplementary material for: Booster vaccination with Ad26.COV2.S or an Omicron-adapted vaccine in pre-immune hamsters protects against Omicron BA.2
Source: NPJ Vaccines. 2023 Mar 16;8:40. doi: 10.1038/s41541-023-00633-x (PMC10018069; doi:10.1038/s41541-023-00633-x)
Supplement: Supplementary file 1 — Supplemental figures [file 41541_2023_633_MOESM1_ESM.pdf]

	S1 subunit (14-685)																																						S2
	NTD (14-305)																														RBD	CTD (542-685)						
AA #	18	19	20	24	25	26	27	67	69	70	75	76	80	95	138	142	143	144	145	156	157	158	190	211	212	213	i214	215	242-244	246-252	253		547	570	614	655	679	681	
Wuhan-Hu-1	L	T	T	L	D	P	A	A	H	V	G	T	D	T	D	G	V	Y	V	E	F	R	R	N	L	V		D			D		T	A	D	H	N	P	
B.1/ B.1.22	.	.	.	.	.	.	.	.	.	.	.	.	.	.	.	.	.	.	.	.	.	.	.	.	.	.		.	.	.	.		.	.	G	.	.	.	
B.1.1.7	.	.	.	.	.	.	.	.	–	–	.	.	.	.	.	.	.	–	.	.	.	.	.	.	.	.		.	.	.	.		.	D	G	.	.	H	
B.1.351	.	.	.	.	.	.	.	.	.	.	.	.	A	.	.	.	.	.	.	.	.	.	.	.	.	.		G	–	.	.		.	.	G	.	.	.	
P.1	F	.	N	.	.	S	.	.	.	.	.	.	.	.	Y	.	.	.	.	.	.	.	S	.	.	.		.	.	.	.		.	.	G	Y	.	.	
B.1.617.2/ AY.5	.	R	.	.	.	.	.	.	.	.	.	.	.	.	.	D	.	.	.	G	–	–	.	.	.	.		.	.	.	.		.	.	G	.	.	R	
C.37	.	.	.	.	.	.	.	.	.	.	V	I	.	.	.	.	.	.	.	.	.	.	.	.	.	.		.	.	–	N		.	.	G	.	.	.	
BA.1	.	.	.	.	.	.	.	V	–	–	.	.	.	I	.	D	–	–	–	.	.	.	.	–	I	.	EPE	.	.	.	.		K	.	G	Y	K	H	
BA.2	.	I	.	–	–	–	S	.	.	.	.	.	.	.	.	D	.	.	.	.	.	.	.	.	.	G		.	.	.	.		.	.	G	Y	K	H	

	RBD (319-541)																			
AA #	339	371	373	375	376	405	408	417	440	446	452	477	478	484	490	493	496	498	501	505
Wuhan-Hu-1	G	S	S	S	T	D	R	K	N	G	L	S	T	E	F	Q	G	Q	N	Y
B.1/ B.1.22	.	.	.	.	.	.	.	.	.	.	.	.	.	.	.	.	.	.	.	.
B.1.1.7	.	.	.	.	.	.	.	.	.	.	.	.	.	.	.	.	.	.	Y	.
B.1.351	.	.	.	.	.	.	.	N	.	.	.	.	.	K	.	.	.	.	Y	.
P.1	.	.	.	.	.	.	.	T	.	.	.	.	.	K	.	.	.	.	Y	.
B.1.617.2/ AY.5	.	.	.	.	.	.	.	.	.	.	R	.	K	.	.	.	.	.	.	.
C.37	.	.	.	.	.	.	.	.	.	.	Q	.	.	.	S	.	.	.	.	.
BA.1	D	L	P	F	.	.	.	N	K	S	.	N	K	A	.	R	S	R	Y	H
BA.2	D	F	P	F	A	N	S	N	K	.	.	N	K	A	.	R	.	R	Y	H

	S2 subunit (686-1273)													
AA #	701	716	764	796	856	859	950	954	969	981	982	1027	1118	1176
Wuhan-Hu-1	A	T	N	D	N	T	D	Q	N	L	S	T	D	V
B.1/ B.1.22	.	.	.	.	.	.	.	.	.	.	.	.	.	.
B.1.1.7	.	I	.	.	.	.	.	.	.	.	A	.	H	.
B.1.351	V	.	.	.	.	.	.	.	.	.	.	.	.	.
P.1	.	.	.	.	.	.	.	.	.	.	.	I	.	F
B.1.617.2/ AY.5	.	.	.	.	.	.	N	.	.	.	.	.	.	.
C.37	.	.	.	.	.	N	.	.	.	.	.	.	.	.
BA.1	.	.	K	Y	K	.	.	H	K	F	.	.	.	.
BA.2	.	.	K	Y	.	.	.	H	K	.	.	.	.	.

Supplemental table 1. Spike mutations in SARS-CoV-2 challenge stocks compared with Wuhan-Hu-1. The mutations/ deletions in spike are shown compared with the Wuhan-Hu-1 isolate (MN908947; lineage B). N-terminal domain (NTD), receptor-binding domain (RBD), C-terminal domain (CTD), dots indicate no differences to the Wuhan-Hu-1 sequence and deletions are indicated with a dash. B.1.1.7 (Alpha), B.1.351 (Beta), P.1 (Gamma), B.1.617.2/ AY.5 (Delta), C.37 (Lambda), BA.1/ BA.2 (Omicron).

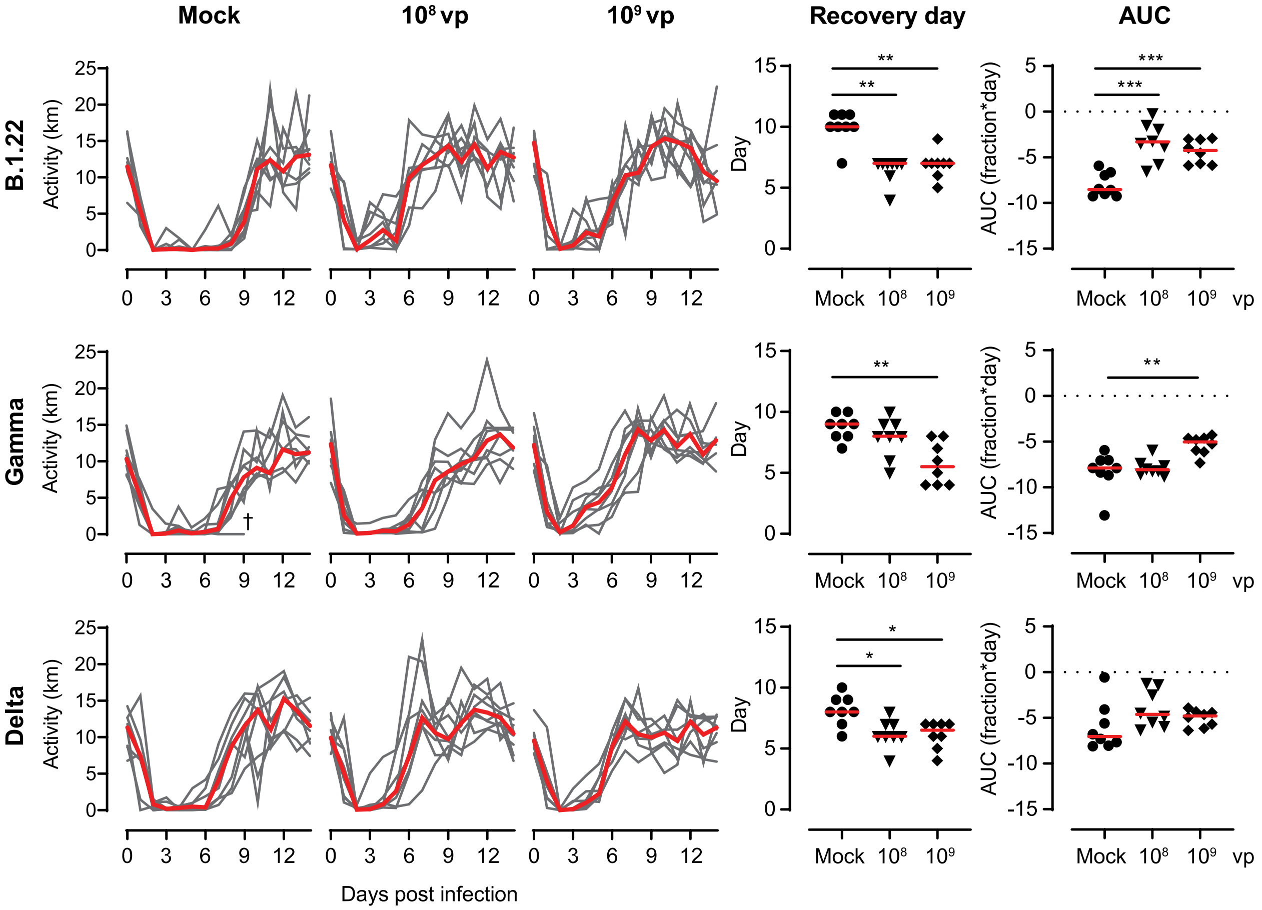

Supplemental figure 1. Ad26.COV2.S protects against activity loss upon SARS-CoV-2 B.1.22, Gamma and Delta challenge. Hamsters were vaccinated with formulation buffer (mock), 10^8 or 10^9 vp Ad26.COV2.S at day -28 (n=8 per group). The animals were intranasally challenged with 10^3 $TCID_{50}$ SARS-CoV-2 B.1.22, 10^4 $TCID_{50}$ SARS-CoV-2 Gamma or 10^4 $TCID_{50}$ SARS-CoV-2 Delta on day 0 and followed up till day 14. Individual activity traces, the day at which animals recovered from activity loss (defined by regaining at least 2.5 km activity a day for 2 consecutive days) and the area under the curve (AUC) as a fraction*day compared with the pre-challenge activity are shown. Red lines indicate group medians. † indicates a humane endpoint was reached. Comparisons to the mock group were performed by ANOVA with a post-hoc t-test or a Mann-Whitney U-test with a 2-fold Bonferroni correction. Statistical differences are indicated by asterisks: * $P<0.05$, ** $P<0.01$, *** $P<0.001$.

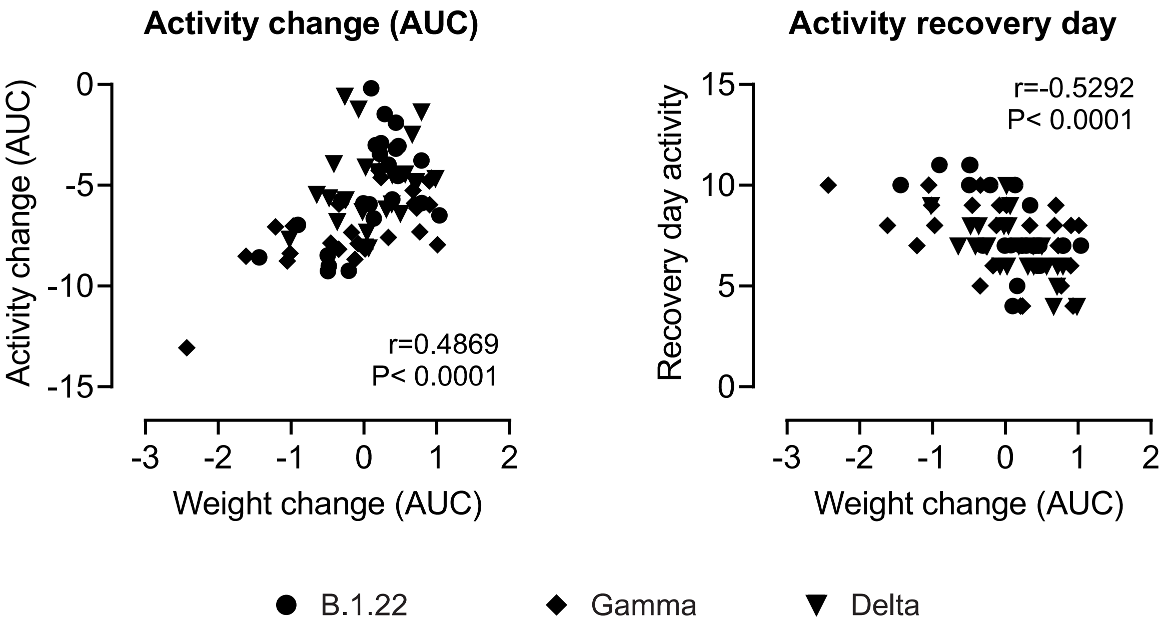

Supplemental figure 2. Correlation activity change with weight change post SARS-CoV-2 challenge. Hamsters were vaccinated with formulation buffer (mock), 10^8 or 10^9 vp Ad26.COV2.S at day -28. Hamsters were intranasally challenged with 10^3 $TCID_{50}$ SARS-CoV-2 B.1.22, 10^4 $TCID_{50}$ SARS-CoV-2 Gamma or 10^4 $TCID_{50}$ SARS-CoV-2 Delta on day 0. The correlation between the area under the curve (AUC as fraction*day compared with pre-challenge weight/ activity) for weight change with the AUC for weight change or the recovery day post-activity change is shown. The day at which animals recovered from activity loss was defined as regaining at least 2.5 km activity a day for 2 consecutive days. Correlation coefficients were calculated across all 3 VOC using two-sided Spearman rank correlation.

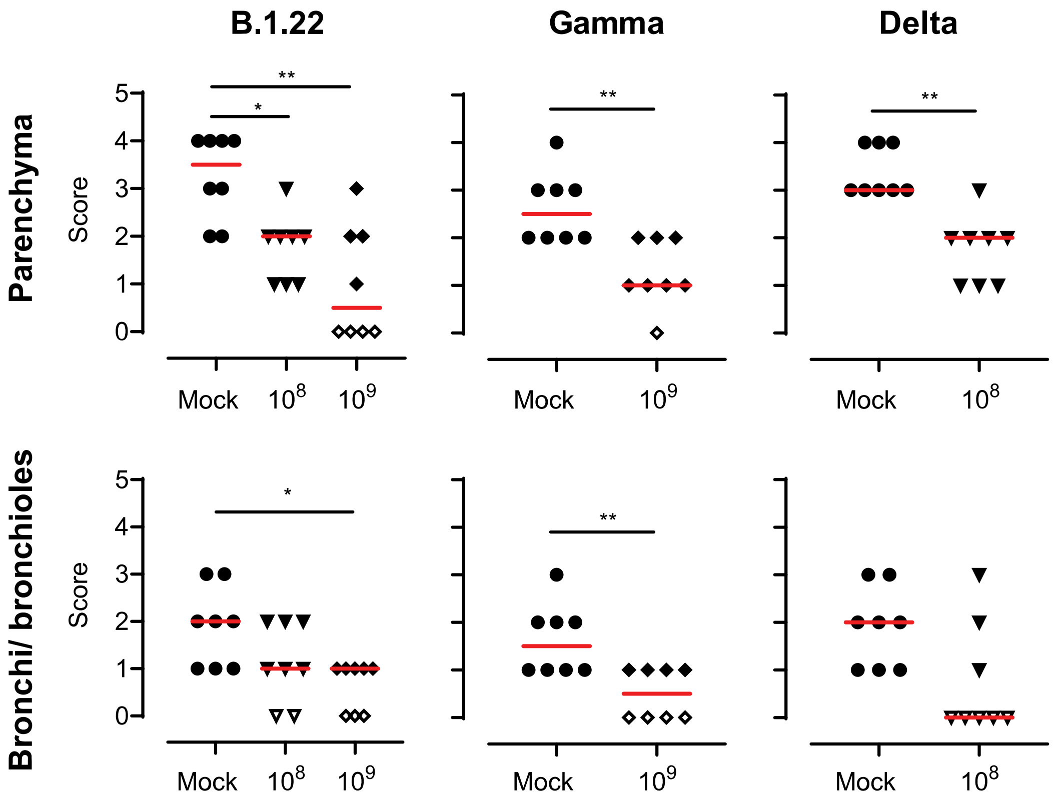

Supplemental figure 3. Nucleocapsid positivity in hamsters after SARS-CoV-2 B.1.22, Gamma and Delta challenge. Hamsters were vaccinated with formulation buffer (mock), 10^8 or 10^9 vp Ad26.COV2.S at day -28 (n=8 per group). The animals were intranasally challenged with 10^3 $TCID_{50}$ SARS-CoV-2 B.1.22, 10^4 $TCID_{50}$ SARS-CoV-2 Gamma or 10^4 $TCID_{50}$ SARS-CoV-2 Delta on day 0. While animals vaccinated with 10^8 and 10^9 vp were evaluated at day 4 after challenge with SARS-CoV-2 B.1.22, only animals vaccinated with 10^9 vp were examined at day 4 after challenge with SARS-CoV-2 Gamma and only 10^8 vp-vaccinated animals were evaluated at day 4 after challenge with the Delta variant. Lungs were stained and scored for SARS-CoV-2 nucleocapsid-protein positivity (score 0-5) in lung parenchyma and in bronchi/bronchioles. Red horizontal bars indicate the median response per group. Open symbols indicate a score of 0. Comparisons were performed by a Mann-Whitney U-test with a 2-fold Bonferroni correction. Statistical differences are indicated by asterisks: * $P<0.05$; ** $P<0.01$.

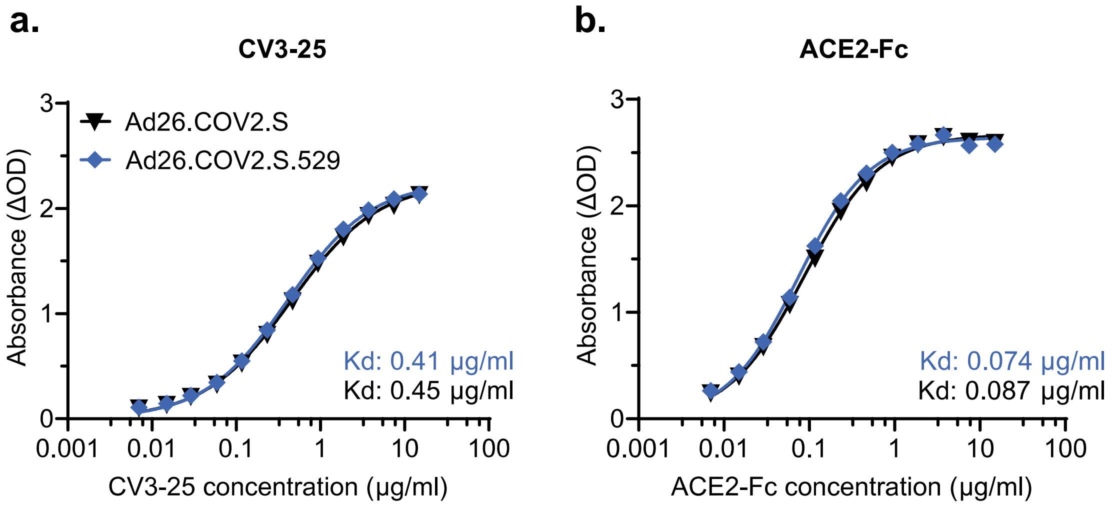

Supplemental figure 4. Comparison of CV3-25 and ACE2-Fc antibody binding to spike after expression by Ad26.COV2.S and Ad26.COV2.S.529. CV3-25 is an antibody that binds to the stem region of the SARS-CoV-2 spike S2[1], which is conserved between the Wuhan-Hu-1 and BA.1 spike protein and ACE2-binding affinity is reported to be similar between Wuhan-Hu-1 Spike and Omicron spike[2,3] **a.** CV3-25 or **b.** ACE2-Fc fusion protein binding to the spike protein in A549 cells after transduction with 2000 infectious units/cell Ad26.COV2.S.529 (blue line) or Ad26.COV2.S (black line). Binding affinity (Kd) values are shown, each point represents the mean value of a duplicate measurement.

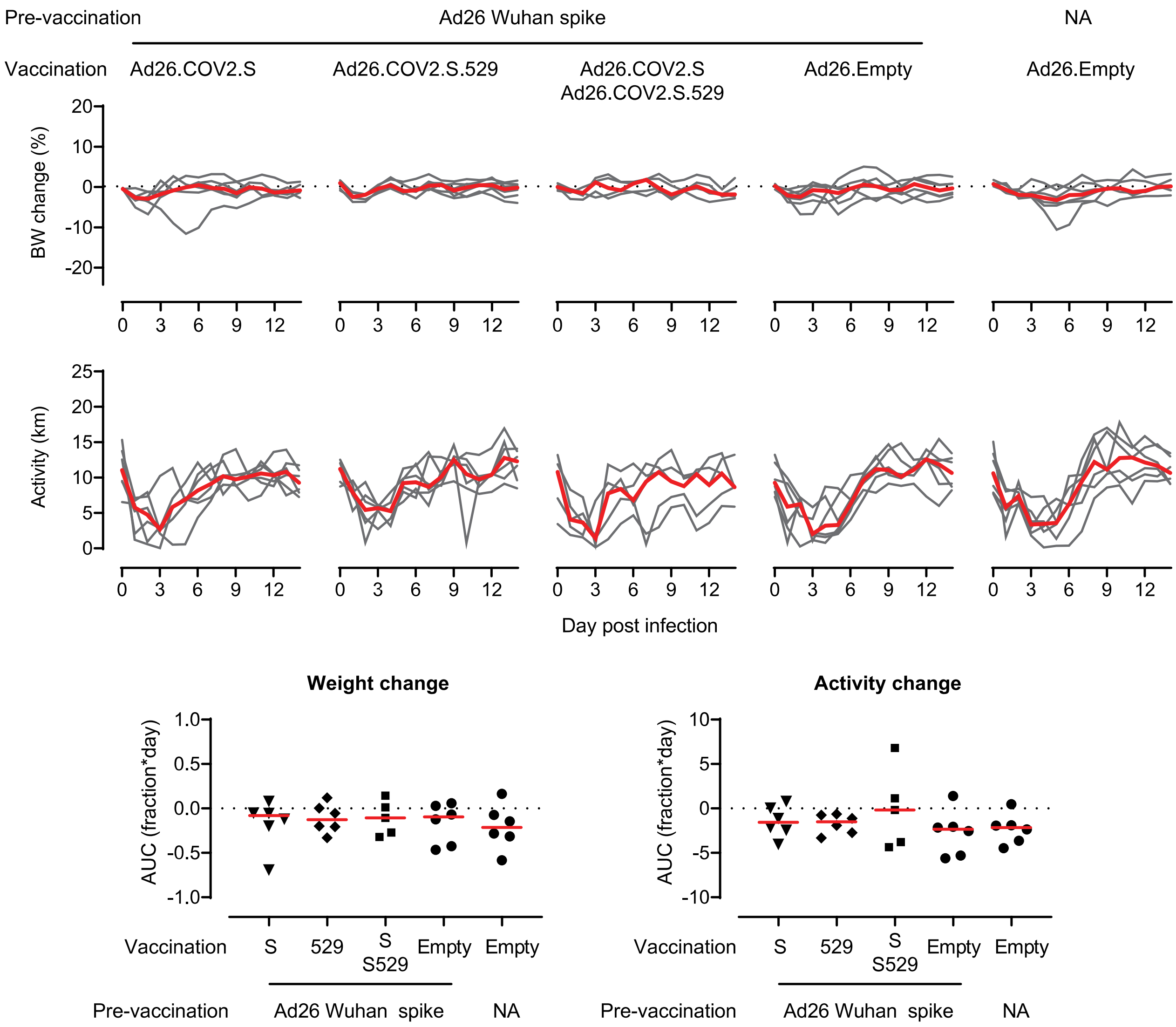

Supplemental figure 5. Weight and activity change after Omicron BA.2 challenge in hamsters. Hamsters with pre-existing immunity were generated by pre-vaccination with 10^7 viral particles (vp) of Ad26NCOV006 (Ad26 vector encoding Wuhan-Hu-1 spike, pre-immune animals) at week -27. Naïve control hamsters were not vaccinated (NA). At week -4, Ad26 Wuhan-Hu-1 spike-vaccinated hamsters were vaccinated with 10^9 vp Ad26.COV2.S (S), Ad26.COV2.S.529 (529), a 1:1 combination of S and 529 or Ad26.Empty (all n=5-6). In addition, naïve hamsters were vaccinated with Ad26.Empty (n=6). The animals were intranasally challenged with $10^{4.7}$ $TCID_{50}$ SARS-CoV-2 BA.2 in week 0. Individual weight and activity traces and the area under the curve (AUC) as a fraction compared with the pre-challenge weight/ activity are shown. Red lines indicate group medians. Comparisons to the pre-vaccinated-only and pairwise comparisons were performed by a t-test with a 3-fold Bonferroni correction. No significant differences were found.

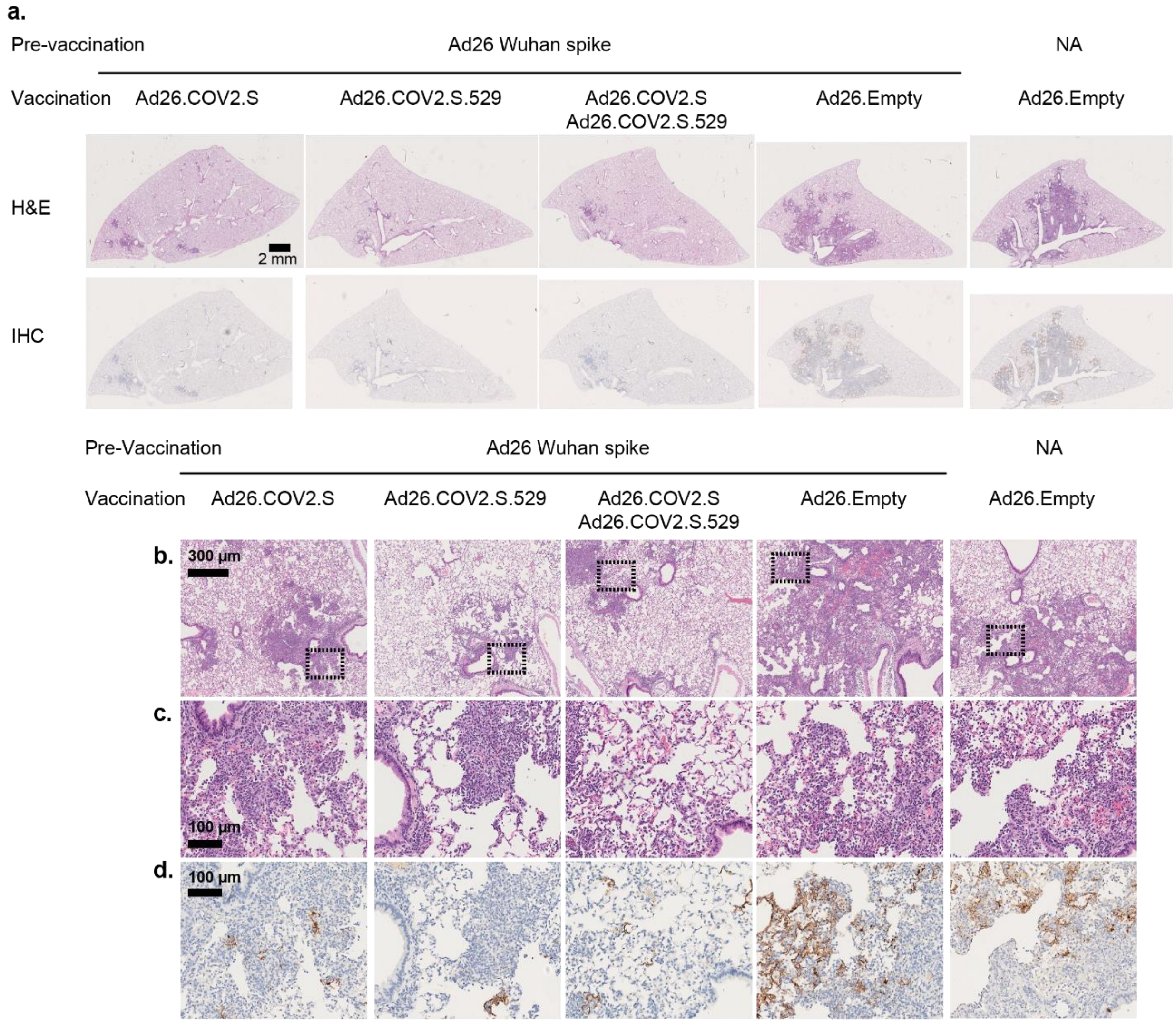

Supplemental figure 6. Representative histopathology images after Omicron BA.2 challenge. Hamsters with pre-existing immunity were generated by pre-vaccination with 10^7 viral particles (vp) of Ad26NCOV006 (Ad26 vector encoding Wuhan-Hu-1 spike, pre-immune animals) at week -27. Naïve control hamsters were not vaccinated (NA). At week -4, Ad26 Wuhan-Hu-1 spike-vaccinated hamsters were vaccinated with 10^9 vp Ad26.COV2.S (S), Ad26.COV2.S.529 (529), a 1:1 combination of S and 529 or Ad26.Empty (all n=8). In addition, naïve hamsters were vaccinated with Ad26.Empty (n=8). The animals were intranasally challenged with $10^{4.7}$ $TCID_{50}$ SARS-CoV-2 BA.2 on week 0. **a.** Representative overview of H&E-stained left lung lobes, and SARS-CoV-2 nucleocapsid positivity visualized using immunohistochemistry (IHC, brown staining). **b.** Representative H&E-stained lung images are shown per group at a 4x magnification. **c.** A 20x magnification of the H&E staining in the rectangular box is shown. **d.** SARS-CoV-2 nucleocapsid positivity shown for the rectangular box in **b** at a 20x magnification.

Supplemental references

1. Jennewein, M. F. *et al.* Isolation and characterization of cross-neutralizing coronavirus antibodies from COVID-19+ subjects. *Cell Rep.* **36**, 109353 (2021).
2. Han, P. *et al.* Receptor binding and complex structures of human ACE2 to spike RBD from omicron and delta SARS-CoV-2. *Cell* **185**, 630-640.e10 (2022).
3. Mannar, D. *et al.* SARS-CoV-2 Omicron variant: Antibody evasion and cryo-EM structure of spike protein–ACE2 complex. *Science* **375**, 760–764 (2022).
