# Supplementary material for: Booster vaccination with Ad26.COV2.S or an Omicron-adapted vaccine in pre-immune hamsters protects against Omicron BA.2
Source: NPJ Vaccines. 2023 Mar 16;8:40. doi: 10.1038/s41541-023-00633-x (PMC10018069; doi:10.1038/s41541-023-00633-x)
Supplement: Supplementary file 2 — REPORTING SUMMARY [file 41541_2023_633_MOESM2_ESM.pdf]

nature portfolio

Corresponding author(s): Roland Zahn

Last updated by author(s): Jan 27, 2023

Reporting Summary

Nature Portfolio wishes to improve the reproducibility of the work that we publish. This form provides structure for consistency and transparency in reporting. For further information on Nature Portfolio policies, see our Editorial Policies and the Editorial Policy Checklist.

Statistics

For all statistical analyses, confirm that the following items are present in the figure legend, table legend, main text, or Methods section.

n/a	Confirmed	
☐	☒	The exact sample size (*n*) for each experimental group/condition, given as a discrete number and unit of measurement
☐	☒	A statement on whether measurements were taken from distinct samples or whether the same sample was measured repeatedly
☐	☒	The statistical test(s) used AND whether they are one- or two-sided<br>*Only common tests should be described solely by name; describe more complex techniques in the Methods section.*
☒	☐	A description of all covariates tested
☐	☒	A description of any assumptions or corrections, such as tests of normality and adjustment for multiple comparisons
☐	☒	A full description of the statistical parameters including central tendency (e.g. means) or other basic estimates (e.g. regression coefficient) AND variation (e.g. standard deviation) or associated estimates of uncertainty (e.g. confidence intervals)
☐	☒	For null hypothesis testing, the test statistic (e.g. *F*, *t*, *r*) with confidence intervals, effect sizes, degrees of freedom and *P* value noted<br>*Give P values as exact values whenever suitable.*
☒	☐	For Bayesian analysis, information on the choice of priors and Markov chain Monte Carlo settings
☒	☐	For hierarchical and complex designs, identification of the appropriate level for tests and full reporting of outcomes
☒	☐	Estimates of effect sizes (e.g. Cohen's *d*, Pearson's *r*), indicating how they were calculated

Our web collection on statistics for biologists contains articles on many of the points above.

Software and code

Policy information about availability of computer code

Data collection	No software was used
Data analysis	SAS 9.4 and GraphPad Prism 9.4.1

For manuscripts utilizing custom algorithms or software that are central to the research but not yet described in published literature, software must be made available to editors and reviewers. We strongly encourage code deposition in a community repository (e.g. GitHub). See the Nature Portfolio guidelines for submitting code & software for further information.

Data

Policy information about availability of data

All manuscripts must include a data availability statement. This statement should provide the following information, where applicable:

- Accession codes, unique identifiers, or web links for publicly available datasets
- A description of any restrictions on data availability
- For clinical datasets or third party data, please ensure that the statement adheres to our policy

All data that support the findings of this study are available from the corresponding author upon reasonable request.

Human research participants

Policy information about studies involving human research participants and Sex and Gender in Research.

Reporting on sex and gender	NA
Population characteristics	NA
Recruitment	NA
Ethics oversight	NA

Note that full information on the approval of the study protocol must also be provided in the manuscript.

Field-specific reporting

Please select the one below that is the best fit for your research. If you are not sure, read the appropriate sections before making your selection.

☒ Life sciences ☐ Behavioural & social sciences ☐ Ecological, evolutionary & environmental sciences

For a reference copy of the document with all sections, see nature.com/documents/nr-reporting-summary-flat.pdf

Life sciences study design

All studies must disclose on these points even when the disclosure is negative.

Sample size	Each study had a predefined objective, and based on historical data obtained in-house, sample sizes were calculated by statisticians to detect predetermined effect sizes with 80% power.
Data exclusions	No data were excluded
Replication	All experiments included either control groups or control time-points validating the experiments. All experiments met the pre-set gatekeepers for the validity of the experiment. Experiments were not replicated due to ethical reasons of animal experimentation
Randomization	allocation was random
Blinding	Operators were blinded to the objectives of the study

Reporting for specific materials, systems and methods

We require information from authors about some types of materials, experimental systems and methods used in many studies. Here, indicate whether each material, system or method listed is relevant to your study. If you are not sure if a list item applies to your research, read the appropriate section before selecting a response.

Materials & experimental systems

n/a	Involved in the study	
☐	☒	Antibodies
☒	☐	Eukaryotic cell lines
☒	☐	Palaeontology and archaeology
☐	☒	Animals and other organisms
☒	☐	Clinical data
☒	☐	Dual use research of concern

Methods

n/a	Involved in the study	
☒	☐	ChIP-seq
☒	☐	Flow cytometry
☒	☐	MRI-based neuroimaging

Antibodies

Antibodies used	CV3-25 antibody (ImmunoPrecise)
Validation	CV3-25 binds the S2 subunit of the SARS-CoV-2 Spike protein [Jennewein, M. F. et al. Isolation and characterization of cross-neutralizing coronavirus antibodies from COVID-19+ subjects. Cell Rep. 36, 109353 (2021)]

Animals and other research organisms

Policy information about studies involving animals; ARRIVE guidelines recommended for reporting animal research, and Sex and Gender in Research

Laboratory animals	Female BALB/c mice aged 8-10 weeks and Female Syrian golden RjHan:AURA hamsters, aged 9-14 weeks
Wild animals	NA
Reporting on sex	Only female hamsters were used in these studies to reduce inter-individual variation, thereby limiting the number of animals required
Field-collected samples	NA
Ethics oversight	Animal experiments were approved by the Central Authority for Scientific Procedures on Animals (Centrale Commissie Dierproeven), the institutional animal welfare body, and conducted in accordance with the European guidelines (EU directive on animal testing 2010/63/EU and ETS 123) and local Dutch legislation on animal experiments

Note that full information on the approval of the study protocol must also be provided in the manuscript.
